# Determinants of a decline in a nutrition risk measure differ by baseline high nutrition risk status: targeting nutrition risk screening for frailty prevention in the Canadian Longitudinal Study on Aging (CLSA)

**DOI:** 10.17269/s41997-023-00745-w

**Published:** 2023-03-22

**Authors:** Heather H. Keller, Vanessa Trinca

**Affiliations:** 1 Department of Kinesiology and Health Sciences, University of Waterloo, Waterloo, Ontario Canada; 2 Schlegel-UW Research Institute for Aging, Waterloo, Ontario Canada

**Keywords:** CLSA, Nutrition status, Aged, Social determinants of health, Frailty, Regression analysis, ELCV, état nutritionnel, personnes âgées, déterminants sociaux de la santé, fragilité, analyse de régression

## Abstract

**Objectives:**

Nutrition risk is a key component of frailty and screening, and treatment of nutrition risk is part of frailty management. This study identified the determinants of a 3-year decline in nutrition risk (measured by SCREEN-8) for older adults stratified by risk status at baseline.

**Methods:**

Secondary data analysis of the comprehensive cohort sample of the Canadian Longitudinal Study on Aging (CLSA) (*n* = 5031) with complete data for covariates at baseline and 3-year follow-up. Using a conceptual model to define covariates, determinants of a change in nutrition risk score as measured by SCREEN-8 (lower score indicates greater risk) were identified for those not at risk at baseline and those at high risk at baseline using multivariable regression.

**Results:**

Models stratified by baseline nutrition risk were significant. Notable factors associated with a decrease in SCREEN-8 for those not at risk at baseline were mental health diagnoses (− 0.83; CI [− 1.44,  −0.22]), living alone at follow-up (− 1.98; CI [− 3.40,  −0.56]), and lack of dental care at both timepoints (− 0.91; CI [− 1.62,  −0.20]) and at follow-up only (− 1.32; CI [− 2.45,  −0.19]). For those at high nutrition risk at baseline, decline in activities of daily living (− 2.56; CI [− 4.36,  −0.77]) and low chair-rise scores (− 1.98; CI [− 3.33, − 0.63]) were associated with lower SCREEN-8 scores at follow-up.

**Conclusion:**

Determinants of change in SCREEN-8 scores are different for those with no risk and those who are already at high risk, suggesting targeted approaches are needed for screening and treatment of nutrition risk in primary care.

**Supplementary Information:**

The online version contains supplementary material available at 10.17269/s41997-023-00745-w.

## Résumé

**Objectifs:**

Le risque nutritionnel est l’un des principaux éléments de la fragilité et le dépistage, et le traitement du risque nutritionnel font partie de la prise en charge de la fragilité. Dans cette étude, nous avons cerné les déterminants du risque de déclin nutritionnel sur 3 ans chez les personnes âgées (mesuré par l’instrument SCREEN-8 – Seniors in the Community Risk Evaluation for Eating and Nutrition), stratifiés selon l’état de risque au départ.

**Méthode:**

Nous avons analysé les données secondaires d’un échantillon de la cohorte globale de l’Étude longitudinale canadienne sur le vieillissement (ELCV) (n = 5 031) avec des données complètes pour les covariables au départ et au suivi après 3 ans. En utilisant un modèle théorique pour définir les covariables, nous avons cerné les déterminants d’un changement du score de risque nutritionnel tel que mesuré par l’instrument SCREEN-8 (un faible score indiquant un risque élevé) pour les personnes qui n’étaient pas à risque au départ et pour celles qui étaient à risque élevé au départ, en faisant appel à la régression multivariée.

**Résultats:**

Les modèles stratifiés selon le risque nutritionnel au départ étaient significatifs. Les facteurs notables associés à la baisse du score SCREEN-8 chez les personnes qui n’étaient pas à risque au départ étaient les diagnostics de santé mentale (-0,83; IC[-1,44, -0,22]), le fait de vivre seul au suivi (-1,98; IC[-3,40, -0,56]) et l’absence de soins dentaires au départ et au suivi (-0,91; IC[-1,62, -0,20]) ou seulement au suivi (-1,32; IC[-2,45, -0,19]). Chez les personnes ayant un risque nutritionnel élevé au départ, la baisse des activités dans la vie quotidienne (-2,56; IC[-4,36, -0,77]) et un faible score pour se lever d’une chaise (-1,98; IC[-3,33, -0,63]) étaient associés à la baisse du score SCREEN-8 au suivi.

**Conclusion:**

Les déterminants d’un changement du score SCREEN-8 sont différents chez les personnes qui ne sont pas à risque au départ et chez celles qui sont déjà à risque élevé, ce qui indique que des approches ciblées sont nécessaires pour le dépistage et le traitement du risque nutritionnel dans les soins primaires.

## Introduction

The World Health Organization (WHO) has dedicated 2021–2030 towards the goal of “healthy aging in older adults” (World Health Organization [WHO], 2020). An area for action is improving integrated care, including through primary care to prevent functional disability and frailty. WHO has called for longitudinal research to support the understanding of healthy aging, as well as policy development and evaluation of interventions (WHO, 2020). Older adults who are frail experience a decline in function and physiological systems, and do not fully recover when a stressor occurs, such as an acute illness (Pilotto et al., 2020; Laur et al., 2017). Screening and assessment are encouraged to identify frailty early and provide interventions (Lee et al., 2018; Pilotto et al., 2020). Food intake (Huang et al., 2021; Khor et al., 2021), anorexia of aging (Merchant et al., 2021), and malnutrition (Rodríguez-Mañas et al., 2021; Laur et al., 2017) are associated with frailty, and screening for nutrition risk and malnutrition is recommended for those who are potentially frail (Lee et al., 2018; Pilotto et al., 2020).

Nutrition(al) risk, although a nebulous term (Bales, 2001), can be used to describe the presence of risk factors and determinants such as food insecurity, poor appetite, and low food intake which could lead to malnutrition (Keller, 2007; Teitelbaum et al., 2005). Malnutrition is demonstrated by changes in body composition (e.g., muscle mass) and body mass that result in functional deficits (e.g., immunity, strength, tolerance, cognition) associated with a nutritional intake that does not meet the body’s needs; this could be due to inadequate food and fluid intake, high metabolic demand, or inability of the body to use the nutrients and energy provided (Cederholm et al., 2017). Although nutrition risk precedes malnutrition in community-living older adults, it is a relevant target for intervention as nutrition risk has been associated with increased use of healthcare resources, and specifically hospital use (Martínez-Reig et al., 2018; Ramage-Morin et al., 2017; Hamirudin et al., 2016). Determinants of food intake (e.g., dysphagia, eating challenges, financial resources), food intake that is inadequate or of poor quality, as well as weight change and other anthropometric indicators should be included on nutrition risk screening tools for community-living older adults as they are early markers of potential malnutrition (Keller, 2007). SCREEN-8 (previously known as SCREEN-IIAB) is a valid and reliable measure of nutrition risk in community-living older adults (Keller et al., 2005). Screening with this tool has the potential to identify nutrition risk before participants lose capacity and become pre-frail or frail. About one third of community-living older Canadians have nutrition risk as per SCREEN-8 (Ramage-Morin et al., 2017).

Nutrition risk and malnutrition more specifically have been described as a “silent threat” that develops in the community (Hamirudin et al., 2016 p. 9) but is often not recognized until a healthcare event such as hospitalization occurs (Allard et al., 2016). Despite the recognition of the importance of malnutrition to frailty (Pilotto et al., 2020), prevention of inadequate food intake and malnutrition in the community is limited (Merchant et al., 2021). To promote healthy aging and reduce malnutrition and frailty, it is recommended that nutrition risk in older adults be identified through screening programs in primary care and community services (Keller et al., 2021; Hamirudin et al., 2016). Understanding individual determinants of nutrition risk is needed to identify further paths for intervention and to mitigate malnutrition in older adults (Volkert et al., 2019).

**Fig. 1:**
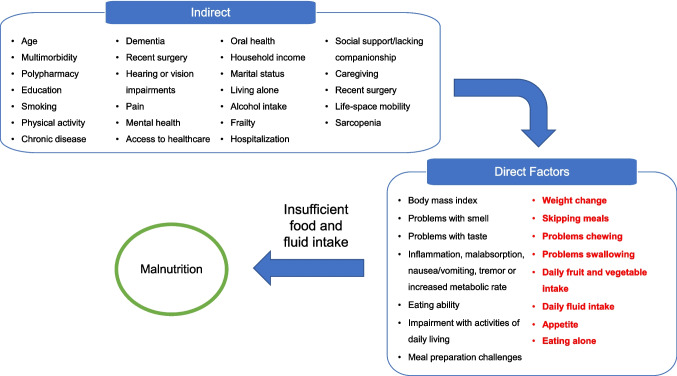
Indirect and direct factors from the Determinants of Malnutrition in Aged Persons (DoMAP) Model available in the Canadian Longitudinal Study on Aging Dataset that may contribute to malnutrition via the low food intake mechanism. Figure adapted from Volkert et al. (2019). Bolded items in red are assessed by the SCREEN-8 tool

Examination of factors associated with nutrition risk in community-living older adults is relatively rare (Hamirudin et al., 2016; Volkert et al., 2019). Most research to date has been based on small samples and cross-sectional data with few factors modelled, limiting our understanding of determinants that are most relevant to target for intervention (Hamirudin et al., 2016). The Determinants of Malnutrition in Aged Persons (DoMAP) is a consensus-based model that identifies diverse sociodemographic, function, and health factors specific to older adults that are associated with malnutrition (Volkert et al., 2019). This model was used to guide our longitudinal analysis to identify determinants of nutrition risk in older adults (≥ 65 years) in the Canadian Longitudinal Study on Aging (CLSA). Figure 1 includes the indirect and direct factors that may contribute to malnutrition adapted from the DoMAP model (Volkert et al., 2019). Analyses were stratified by baseline participant nutrition risk, to understand the determinants of risk and the determinants that perpetuate continued nutrition risk, potentially leading to malnutrition and frailty. This study aimed to identify (1) what proportion of community-dwelling older adults experienced a change in nutrition risk over a 3-year period and if the change differed by baseline risk stratification, and (2) what health, function, sociodemographic, and healthcare service use variables are associated with 3-year change in nutrition risk for older persons stratified by baseline risk.

## Methods

### Sample

The CLSA is the largest and most comprehensive Canadian prospective cohort study assessing longitudinal data regarding aging. Greater description of the CLSA has been published previously (Raina et al., 2009). Every 3 years, participants complete data collection for a minimum of 20 years, or until death. Only data from participants in the comprehensive cohort (baseline *n* = 30,097) were used in this analysis. Data collection occurred through questionnaires, physical examination, and biological samples. Participants were recruited from areas within a 25–50 km radius of 11 major academic centres in Canada, representing 4 regions: Pacific Coast, the Prairies, Central Canada, and the Atlantic Region. The CLSA included participants with a minimum age of 45 at baseline. Participants were excluded if they were residing in the three territories, some remote regions, federal First Nations reserves, and other First Nations settlements in the provinces, were full-time members of the Canadian Armed Forces, and/or were living in institutions (e.g., long-term care homes) at baseline; older adults living in settings where minimal care is provided (e.g., retirement homes) were included. Participants had to be able to respond in English or French and have no cognitive impairment at baseline. Informed consent was obtained from all participants. Baseline data collection occurred during 2012–2015 and first follow-up during 2015–2018. For our analysis, we included participants who were  ≥65 at baseline (*n* = 12,646) and excluded participants who reported current abdominal or nasogastric tube feeding, resulting in an initial eligible sample of 12,630. Ethics review was completed by the University of Waterloo (ORE#42598).

### Variables used

Nutrition risk was assessed using the valid and reliable SCREEN-8 tool (Keller et al., 2005; note rebranded in 2019 to SCREEN-14 (original SCREEN-II) and SCREEN-8 (original SCREEN-IIAB see www.olderadultnutritionscreening.com)). SCREEN-8 asks eight questions regarding weight change, appetite, eating challenges, and fluid and fruit/vegetable intake. The minimum and maximum scores are 0 and 48, respectively. A score of  <38 indicates high nutrition risk, a value previously shown to predict hospitalization and mortality (Ramage-Morin et al., 2017). A positive change when comparing follow-up to baseline indicates a score improvement, while a negative change indicates a decline from baseline.

Variables were selected based on the DoMAP model (Volkert et al., 2019), and included health, social determinants of health, healthcare use, and demographic variables. Both time-independent (i.e., variable collected only at baseline or follow-up) and time-dependent variables (i.e., variable was collected at both baseline and follow-up) were used. Variable selection and categorization were dictated by both clinical/practical relevance and data collection protocols to ensure sufficient distribution within variable groupings to prevent challenges that arise with low cell counts within a variable category. All variables were self-reported by participants, except for body mass index which was measured by trained research staff. A summary of items collected at baseline and follow-up in the CLSA and subsequent categorization are in Table 1.

**Table 1:** Questionnaire items, data collection methods and variable categorization used in descriptive and multivariable statistics

	Question stem	Time collected	Categorization for analysis
**Time-independent variable **			
Sex	Are you male or female?	Baseline	• Female • Male
Age	Age group	Baseline	• 65–69 • 70–74 • 75–79 • ≥80
Current smoker	Do you currently smoke?	Baseline	• Yes • No
Education level	Highest level of education attained	Baseline	• Less than secondary school • Secondary school • Some post-secondary education • Post-secondary degree or diploma
Polypharmacy	Prescription and non-prescription medication taken regularly. Dichotomized based on Varghese et al. (2021)	Baseline	• <5 medications • ≥5 medications
Body mass index	Height and weight measured by research staff Dichotomized based on GLIM criteria (Cederholm et al., 2019) Low: defined as <20 kg/m^2^ for participants <70 years and <22 kg/m^2^ for participants ≥70 years Adequate: defined as ≥20 kg/m^2^ for participants <70 years and ≥22 kg/m^2^ for participants ≥70 years	Baseline	• Low • Adequate
Physical Activity Scale for the Elderly score	Physical Activity Scale for the Elderly (PASE) score considers physical activity type and frequency over the last 7 days. There is no upper limit score. Quartiles were used to categorize levels of physical activity (Washburn et al., 1993)	Baseline	• 0–72.00 • >72.00–110.72 • >110.72–148.43 • >148.43
Social Support Survey score	Measured using the 19-item Medical Outcomes Study Social Support Survey. Score ranges from 0 to 100, with higher scores indicating greater support. Quartiles were used to categorize levels of social support (Sherbourne & Stewart, 1991)	Baseline	• 0–69.74 • > 69.74–84.21 • > 84.21–94.74 • > 94.74
Problems with smell	Do you have problems with the sense of smell (e.g., decreased perception or smelling non-appropriate odours)?	Follow-up	• Yes • No
Problems with taste	Do you have problems tasting foods (e.g., impaired taste for sweet or salty foods or having an unusual sweet, salty, sour, or bitter taste in the mouth)?	Follow-up	• Yes • No
Lacking companionship	How often do you feel that you lack companionship?	Follow-up	• Hardly ever • Some of the time • Often
**Time-dependent variable**			
	Question stem/included items	Categorization at baseline and follow-up	Change variable created for analysis^a^
Dementia/neurological condition	Has a doctor ever told you that you have: memory problems, dementia or Alzheimer’s disease, epilepsy, multiple sclerosis, parkinsonism or Parkinson’s disease	• Yes • No	• No/No • No/Yes, Yes/No, Yes/Yes
Kidney condition	Has a doctor ever told you that you have kidney disease or kidney failure?	• Yes • No	• No/No • No/Yes, Yes/No, Yes/Yes
Mental health condition	Has a doctor ever told you that you have (a): mood disorder, anxiety disorder, clinical depression	• No • Yes	• No/No • No/Yes • Yes/No • Yes/Yes
Cancer	Has a doctor ever told you that you have cancer?	• No • Yes	• No/No • Yes/No, Yes/Yes • No/yes
Gastrointestinal condition	Has a doctor ever told you that you have a bowel disorder, intestinal or stomach ulcers	• No • Yes	• No/No • Yes/No, Yes/Yes • No/Yes
Cardiovascular condition	Has a doctor ever told you that you have: angina, high blood pressure/hypertension, heart disease, peripheral vascular disease or poor circulation in limbs, experienced a stroke/cerebrovascular accident, ministroke/transient ischemic attack?	• No • Yes	• No/No • Yes/No, Yes/Yes • No/Yes
Osteoporosis	Has a doctor ever told you that you have osteoporosis?	• No • Yes	• No/No • Yes/No, Yes/Yes • No/Yes
Endocrine condition	Has a doctor ever told you that you have: diabetes, borderline diabetes or high blood sugar, under- or over-active thyroid gland?	• No • Yes	• No/No • Yes/No, Yes/Yes • No/Yes
Arthritis^b^	Has a doctor ever told you that you have osteoarthritis in one or both hands, hip, or knee, rheumatoid arthritis or another type of arthritis?	• No • Yes	• No/No • Yes/No, Yes/Yes • No/Yes
Respiratory condition	Has a doctor ever told you that you have asthma, emphysema, chronic bronchitis, chronic obstructive pulmonary disease, or chronic changes in lungs due to smoking?	• No • Yes	• No/No • Yes/No, Yes/Yes • No/Yes
Incontinence	Has a doctor ever told you that you have urinary incontinence or bowel incontinence?	• No • Yes	• No/No • Yes/No, Yes/Yes • No/Yes
Recent surgery	Have you had a surgery within the last 3 months?	• No • Yes	• No/No • Yes/No, Yes/Yes • No/yes
Pain-free rating	Are you usually free of pain or discomfort?	• No • Yes	• No/No • No/Yes • Yes/No • Yes/Yes
Self-rated hearing	Is your hearing, using a hearing aid if you use one…	• Excellent • Very good • Good • Fair • Poor	• No change • Increase from baseline • Decrease from baseline
Self-rated vision	Is your eyesight, using glasses or corrective lens if you use them…	• Excellent • Very good • Good • Fair • Poor	• No change • Increase from baseline • Decrease from baseline
Self-rated general health	In general, would you say your health is…	• Excellent • Very good • Good • Fair • Poor	• No change • Increase from baseline • Decrease from baseline
Self-rated mental health	In general, would you say your mental health is …	• Excellent • Very good • Good • Fair • Poor	• No change • Increase from baseline • Decrease from baseline
Psychologist/social service use	During the past 12 months, have you had contact with a psychologist or social worker about your physical or mental health?	• No • Yes	• No/No • No/Yes, Yes/No, Yes/Yes
General practitioner/family physician visit	During the past 12 months, have you had contact with a general practitioner/family physician about your physical or mental health?	• No • Yes	• No/No, No/Yes, Yes/No • Yes/Yes
Allied healthcare use	During the past 12 months, have you had contact with a physiotherapist, occupational therapist, or chiropractor about your physical or mental health?	• No • Yes	• No/No • No/Yes • Yes/No • Yes/Yes
Ophthalmologist/optometrist visit	During the past 12 months, have you had contact with an ophthalmologist/optometrist about your physical or mental health?	• No • Yes	• No/No • No/Yes • Yes/No • Yes/Yes
Dentist visit	During the past 12 months, have you had contact with a dentist about your physical or mental health?	• No • Yes	• No/No • No/Yes • Yes/No • Yes/Yes
Hospital service use	During the past 12 months, have you been seen in an emergency department, or been a patient in a hospital overnight for your physical or mental health?	• No • Yes	• No/No • No/Yes • Yes/No • Yes/Yes
Household income	What is your best estimate of the total household income received by all household members, from all sources, before taxes and deductions, in the past 12 months?	• <$20,000 • $20,000–$49,999 • $50,000–$99,999 • $100,000–$149,999 • ≥$150,000	• No change • Decrease from baseline • Increase from baseline
Marital status	What is your current marital/partner status?	• Single, never married, or never lived with a partner • Married/living with a partner in a common-law relationship • Widowed • Divorced • Separated	• No change • Any change
Living alone	Number of people living in household (excluding the participant). If the participant was living with one or more persons this was coded as “No”	• No • Yes	• No/No • Yes/No • No/Yes • Yes/Yes
Alcohol intake frequency	Frequency of alcohol consumption in the past 12 months	• Almost daily • 4–5 times a week • 2–3 times a week • Once a week • 2–3 times a month • About once a month • Less than once a month • Never	• No change • Decrease from baseline • Increase from baseline
Help required to prepare meals/meal delivery	During the past 12 months, did you receive short- or long-term professional or non-professional assistance with meal preparation because of a health condition or limitation that affects your daily life?	• No • Yes	• No/No • Yes/No, Yes/Yes, No/Yes
Impairment with activities of daily living	Measured using modified questions from the Older Americans Resources and Services Multidimensional Functional Assessment Questionnaire (Fillenbaum & Smyer, 1981). Scores were determined from 7 activities of daily living and 7 instrumental activities of daily living. Dichotomized as no impairment, or mild to total impairment	• No • Yes	• No/No • No/Yes • Yes/No • Yes/Yes
Chair rise time	Total time required to complete chair rise (in seconds) ≤15 s vs. >15 s selected based on Cruz-Jentoft et al., 2019	• ≤15 s (adequate performance) • >15 s (poor performance)	• ≤15 s/≤15 s • ≤15 s/>15 s • >15 s/≤15 s • >15 s/>15 s
Life Space Index score	The Life Space Index score asks participants about their mobility and frequency in the home and community (Baker et al., 2003). Previous work in the CLSA identified that a clinically meaningful change in score is indicated by changes >5 points (Kennedy et al., 2019)	• |Follow-up – Baseline score| > 5	• No change • Increase from baseline • Decrease from baseline
Oral health problems	• In the past 12 months, have you experienced: problems chewing, burning mouth, bad breath, jaw, natural tooth or denture issues, or swelling in the mouth? • In the past 12 months, how often have you: avoided eating particular foods or have you found it uncomfortable to eat any food because of problems with your mouth?	• Yes • No - This was coded as “No” when scored as “never” or “rarely”, and “Yes” when scored as “sometimes” or “often”	• No/No • No/Yes • Yes/No • Yes/Yes
Multimorbidity	Number of chronic conditions. Dichotomized based on Salive, 2013	• <2 • ≥2	• <2/<2 • <2/≥2 • ≥2/<2 • ≥2/≥2
Caregiver status	Respondent provided assistance in the past 12 months to another person (outside of work/volunteering) because of a health condition or limitation	• No • Yes	• No/No • No/Yes • Yes/No • Yes/Yes

^a^When a “/” is used to separate baseline and follow-up measure. For example, “No/Yes” indicates that the variable of interest was not present at baseline, but present at follow-up

^b^At follow-up, the diagnosis module had the same number of responses for all variables (e.g., dementia, ulcer, anxiety disorder) except for rheumatoid arthritis. Rheumatoid arthritis was coded as missing for 3029 participants in the original sample (*n* = 12,636); however, they had completed all other variables in the diagnosis module. If data for the diagnosis section were missing for all diagnoses, then rheumatoid arthritis was also coded as missing. If all diagnoses in the diagnosis module were completed except for rheumatoid arthritis, the baseline value was carried forward and imputed at follow-up so sample size was not lost due to this variable

Variables were categorized based on a combination of both clinical relevance and data distribution

### Analysis

Statistical analyses were performed using SAS Studio® Release 3.81. Descriptive statistics (proportions, mean, SD) were determined. The sample of participants who were excluded due to missing data and those included in the final analytical sample were compared on key demographic variables to determine potential challenges with generalizability of findings using the Rao-Scott likelihood ratio chi-square test. Multivariable linear regression was used to determine associations between all operationalized variables based on the DoMAP model variables available in the CLSA and change in SCREEN-8 score. Survey methodologies were accounted using the SURVEYREG procedure, and analytic and geographic weights were used. Listwise deletion for all variables was used in all analyses, resulting in a final sample of 5031. Statistical significance was determined by *p* < 0.05.

## Results

### Descriptive statistics

Descriptive statistics for baseline and follow-up variables are in Table 2. Key descriptive statistics stratified by baseline nutrition risk status are in Table 3; descriptive statistics stratified by nutrition risk status for all variables are in Supplementary Table 1. Analytic sample participants and those excluded from analyses due to missing data were compared; those excluded were more likely to be female, older, less educated, have a lower income, be widowed, live alone, and be at nutrition risk (see Supplementary Table 2). Statistical significance between risk groups was found for many of the personal characteristics of participants. At baseline, 71.0% of participants were not at high nutrition risk, while 64.4% were not at high risk at follow-up. Of the 5031 participants, 53.6% remained not at nutrition risk at both baseline and follow-up, and 18.3% remained at nutrition risk at both timepoints. Meanwhile, 28.0% of participants experienced a change in nutrition risk status, with 17.3% experiencing a decline (i.e., not at risk at baseline, at risk at follow-up) and 10.7% an improvement (i.e., at risk at baseline, not at risk at follow-up). Mean change in SCREEN-8 score was  −1.03 (SD 5.39; median =  − 1.00). Among the 3570 participants not at high nutrition risk at baseline, change in SCREEN-8 score was  −2.20 (SD 4.63; median =  − 2.00), and among the 1461 participants at high nutrition risk at baseline, change in SCREEN-8 score was +1.82 (SD 6.02; median = 2.00).

**Table 2:** Descriptive statistics for baseline and follow-up variables (*n* = 5031)

		Baseline		Follow-up	
		%	*n*	%	*n*
Sex	Female	44.8	2252	-	-
	Male	55.2	2779	-	-
Age	65–69	41.0	2062	-	-
	70–74	26.0	1306	-	-
	75–79	22.9	1154	-	-
	≥80	10.1	509	-	-
Body mass index	Adequate	94.4	4747	-	-
	Low	5.6	284	-	-
Education level	Less than secondary school	6.7	339	-	-
	Secondary school, no post-secondary education	9.6	485	-	-
	Some post-secondary education	7.6	383	-	-
	Post-secondary degree/diploma	76.0	3824	-	-
Household income	<$50,000	33.4	1680	32.7	1643
	$50,000–$99,999	42.8	2154	43.1	2169
	≥$100,000	23.8	1197	24.2	1219
Marital status	Single, never married or never lived with a partner	4.8	243	5.4	270
	Married/living with a partner in a common-law relationship	68.6	3450	67.0	3371
	Widowed	14.2	713	15.6	783
	Divorced/separated	12.4	625	12.1	607
Living situation	Alone	26.0	1307	28.0	1411
	With one other person	64.3	3237	63.7	3204
	With two or more people	9.7	487	8.3	416
Current smoker	Yes	4.5	229	-	-
	No	95.5	4802	-	-
Alcohol intake frequency in the last year	Never	1.9	95	2.7	135
	Not in the last year	10.2	511	9.1	460
	Less than once a month	10.2	515	10.7	537
	About once a month	6.5	326	7.1	356
	2–3 times a month	9.5	478	9.1	457
	Once a week	10.3	519	10.5	529
	2–3 times a week	18.0	905	17.5	878
	4–5 times a week	9.6	482	9.6	483
	Almost every day	23.9	1200	23.8	1196
Polypharmacy	<5 medications	48.5	2440	-	-
	≥5 medications	51.5	2591	-	-
Multimorbidity	<2 conditions	23.8	1195	17.5	879
	≥2 conditions	76.2	3836	82.5	4152
Social Support Survey score	0–69.74	20.8	1046	-	-
	>69.74–84.21	27.4	1379	-	-
	>84.21–94.74	24.9	1253	-	-
	>94.74	26.9	1353	-	-
Lacking companionship	Hardly ever	-	-	73.4	3695
	Some of the time	-	-	22.1	1113
	Often	-	-	4.4	223
Physical Activity Scale for the Elderly score	0–72.00	18.1	913	-	-
	>72.00–110.72	23.9	1203	-	-
	>110.72–148.43	27.2	1368	-	-
	>148.43	30.7	1547	-	-
Caregiver status	No	57.9	2912	49.5	2492
	Yes	42.1	2119	50.5	2539
Problems with smell	No	-	-	87.8	4419
	Yes	-	-	12.2	612
Problems with taste	No	-	-	93.9	4723
	Yes	-	-	6.1	308
Self-rated hearing	Excellent	18.3	920	9.2	463
	Very good	32.5	1635	30.7	1545
	Good	35.9	1804	41.7	2096
	Fair	11.7	589	16.5	828
	Poor	1.6	83	2.0	99
Self-rated vision	Excellent	22.1	1112	15.9	802
	Very good	39.6	1994	39.6	1993
	Good	31.3	1577	35.8	1799
	Fair	6.0	304	7.5	377
	Poor	0.9	44	1.2	60
Self-rated general health	Excellent	24.8	1247	21.1	1061
	Very good	44.9	2259	44.2	2224
	Good	25.7	1295	27.1	1363
	Fair	4.2	209	6.6	334
	Poor	0.4	21	1.0	49
Self-rated mental health	Excellent	34.0	1709	29.0	1459
	Very good	43.4	2183	43.1	2170
	Good	20.1	1012	24.5	1233
	Fair	2.4	121	3.2	161
	Poor	0.1	6	0.2	8
Dementia/neurological condition	No	97.6	4908	96.6	4862
	Yes	2.4	123	3.4	169
Mental health condition	No	82.8	4167	82.7	4160
	Yes	17.2	864	17.3	871
Cancer	No	78.6	3955	73.4	3694
	Yes	21.4	1076	26.6	1337
Gastrointestinal condition	No	84.5	4252	82.8	4167
	Yes	15.5	779	17.2	864
Cardiovascular condition	No	43.7	2198	39.7	2000
	Yes	56.3	2833	60.3	3031
Osteoporosis	No	86.9	4373	85.0	4278
	Yes	13.1	658	15.0	753
Endocrine condition	No	68.5	3449	64.6	3250
	Yes	31.5	1582	35.4	1781
Arthritis	No	59.9	3015	53.7	2702
	Yes	40.1	2016	46.3	2329
Respiratory condition	No	86.1	4333	84.6	4254
	Yes	13.9	698	15.4	777
Incontinence	No	89.6	4510	76.0	3826
	Yes	10.4	521	24.0	1205
Kidney condition	No	96.7	4863	95.8	4821
	Yes	3.3	168	4.2	210
Surgery in the last 3 months	No	95.1	4783	94.1	4736
	Yes	4.9	248	5.9	295
Pain-free rating	No	33.0	1658	28.1	1416
	Yes	67.0	3373	71.9	3615
Oral health problems	No	50.6	2548	49.2	2474
	Yes	49.4	2483	50.8	2557
Dentist visit	No	18.0	903	13.7	688
	Yes	82.0	4128	86.3	4343
Psychologist/social service use	No	96.7	4864	96.2	4838
	Yes	3.3	167	3.8	193
Allied healthcare use	No	69.6	3502	66.9	3368
	Yes	30.4	1529	33.1	1663
General practitioner/family physician visit	No	6.1	308	5.4	270
	Yes	93.9	4723	94.6	4761
Ophthalmologist/optometrist visit	No	31.0	1560	36.1	1815
	Yes	69.0	3471	63.9	3216
Hospital service use	No	79.5	4000	75.0	3774
	Yes	20.5	1031	25.0	1257
Help required to prepare meals/meal delivery	No	96.7	4867	91.1	4581
	Yes	3.3	164	8.9	450
Impairment with activities of daily living	No	92.0	4629	86.9	4374
	Yes	8.0	402	13.1	657
Chair rise time	≤15 s	66.5	3347	69.4	3489
	>15 s	33.5	1684	30.6	1542
Life Space Index score	Mean (SD)	81.97 (16.53)		84.67 (15.98)	
Nutrition risk status	Not at risk	71.0	3570	64.4	3238
	At risk	29.0	1461	35.6	1793

**Table 3:** Select participant descriptive statistics for all participants and stratified by baseline nutrition risk status

		All (*n* = 5031)		Not at risk (*n* = 3570)		At high risk (*n* = 1461)		Rao-Scott chi-square
Variable		*n*	%	*n*	%	*n*	%	
Sex	Female	2252	44.8	1537	43.1	715	48.9	***χ***^**2**^** = 8.87** ***p* = 0.003**
	Male	2779	55.2	2033	56.9	746	51.1	
Age	65–69	2062	41.0	1467	41.1	595	40.7	*χ*^2^ = 2.84 *p* = 0.420
	70–74	1306	26.0	931	26.1	375	25.7	
	75–79	1154	22.7	809	22.7	345	23.6	
	≥80	509	10.1	363	10.2	146	10.0	
Body mass index	Low	284	94.4	207	5.8	77	5.3	*χ*^2^ = 0.09 *p* = 0.770
	Adequate	4747	5.7	3363	94.2	1384	94.7	
Current smoker	No	4802	95.5	3466	97.1	1336	91.4	***χ***^**2**^** = 19.14** ***p* < 0.001**
	Yes	229	4.5	104	2.9	125	8.6	
Chair rise time	≤15 s/≤15 s (stable adequate)	2726	54.2	1989	55.7	737	50.4	***χ***^**2**^** = 11.20** ***p* = 0.011**
	≤15 s/>15 s (decline)	621	12.3	425	11.9	196	13.4	
	>15 s/≤15 s (improved)	763	15.2	540	15.1	223	15.3	
	>15 s/>15 s (stable poor)	921	18.3	616	17.3	305	20.9	
Education level	Less than secondary school	339	6.7	212	5.9	127	8.7	***χ***^**2**^** = 16.17** ***p* = 0.001**
	Secondary school, no post-secondary education	485	9.6	339	9.5	146	10.0	
	Some post-secondary education	383	7.6	250	7.0	133	9.1	
	Post-secondary degree/diploma	3824	76.0	2769	77.5	1055	72.2	
Household income	No change	3936	78.2	2789	78.1	1147	78.5	*χ*^2^ = 1.16 *p* = 0.561
	Decrease from baseline	515	10.2	369	10.3	146	10.0	
	Increase from baseline	580	11.5	412	11.5	168	11.5	
Marital status	No change	4801	95.4	3432	96.1	1369	93.7	*χ*^2^ = 1.73 *p* = 0.188
	Any change	230	4.6	138	3.9	92	6.3	
Living alone	No/No	3506	69.7	2710	75.9	796	54.5	***χ***^**2**^** = 111.51** ***p* < 0.001**
	Yes/No	114	2.3	67	1.9	47	3.2	
	No/Yes	218	4.3	131	3.7	87	6.0	
	Yes/Yes	1193	23.7	662	18.5	531	36.3	
Activities of daily living	No/No	4177	83.0	3056	85.6	1121	76.7	***χ***^**2**^** = 32.81** ***p* < 0.001**
	No/Impairment	452	9.0	272	7.6	180	12.3	
	Impairment/No	197	3.9	124	3.5	73	5.0	
	Impairment/Impairment	205	4.1	118	3.3	87	6.0	

When a “/” is used to separate baseline and follow-up measure. For example, “No/Yes” indicates that the variable of interest was not present at baseline, but present at follow-up. Bolded terms indicate statistical significance (*p* < 0.05) between the at risk and not at risk groups

### Multivariable regression

Model effects are reported in Table 4 and the odds ratios for only significant model effects are included in Table 5. The full model is included in Supplementary Table 3.

**Table 4:** Model effects testing the association factors with 3-year change in SCREEN-8 score, stratified by baseline nutrition risk status

	Not at risk (*n* = 3570)			At high risk (*n* = 1461)		
Effect	DF	*F*-value	*p*-value	DF	*F*-value	*p*-value
Model	**94**	**2.68**	** <0.001**	**94**	**2.03**	** <0.001**
Intercept	**1**	**55.56**	** <0.001**	1	0.11	0.743
Sex	**1**	**5.70**	**0.017**	1	0.87	0.350
Age	3	2.09	0.099	3	0.28	0.838
Body mass index	1	1.37	0.241	1	0.40	0.529
Education level	3	0.41	0.746	3	0.27	0.849
Household income	2	0.60	0.547	**2**	**4.88**	**0.008**
Marital status	**1**	**5.97**	**0.015**	1	2.03	0.154
Living alone	**3**	**2.90**	**0.034**	3	0.21	0.887
Current smoker	**1**	**5.06**	**0.025**	1	2.90	0.089
Alcohol intake frequency	2	0.04	0.962	2	0.21	0.814
Polypharmacy	1	0.37	0.542	1	0.60	0.438
Multimorbidity	3	0.22	0.882	3	1.56	0.197
Social Support Survey score	3	1.04	0.372	3	0.39	0.763
Lacking companionship	2	0.30	0.740	2	0.74	0.478
Physical Activity Scale for the Elderly score	3	1.99	0.113	3	0.99	0.397
Caregiver status	3	1.54	0.201	3	1.55	0.200
Problems with smell	1	0.77	0.380	1	3.06	0.081
Problems with taste	1	0.12	0.726	1	0.85	0.356
Self-rated hearing	2	0.87	0.420	**2**	**3.49**	**0.031**
Self-rated vision	2	2.41	0.090	2	1.09	0.338
Self-rated general health	2	0.39	0.676	2	2.65	0.071
Self-rated mental health	2	1.02	0.362	2	1.52	0.220
Dementia/neurological condition	1	0.78	0.379	1	0.31	0.579
Mental health condition	**3**	**3.40**	**0.017**	3	2.35	0.071
Cancer	2	1.49	0.225	2	2.31	0.100
Gastrointestinal condition	2	0.76	0.467	**2**	**4.84**	**0.008**
Cardiovascular condition	2	0.78	0.459	2	0.24	0.788
Osteoporosis	2	0.06	0.944	2	0.45	0.638
Endocrine condition	2	0.44	0.644	2	2.25	0.106
Arthritis	2	0.42	0.656	2	0.17	0.842
Respiratory condition	2	2.41	0.090	2	0.52	0.597
Incontinence	2	1.03	0.357	2	1.11	0.329
Kidney condition	**1**	**5.20**	**0.023**	1	0.04	0.833
Surgery in the last 3 months	2	0.91	0.403	2	0.27	0.765
Pain-free rating	3	0.16	0.922	**3**	**3.88**	**0.009**
Oral health problems	**3**	**4.99**	**0.002**	**3**	**2.72**	**0.043**
Dentist visit	**3**	**3.91**	**0.009**	3	2.25	0.080
Psychologist/social service use	**1**	**7.72**	**0.006**	1	1.70	0.193
Allied healthcare use	3	0.22	0.881	3	2.14	0.093
General practitioner/family physician visit	1	1.55	0.213	1	3.78	0.052
Ophthalmologist/optometrist visit	3	1.87	0.132	3	0.70	0.554
Hospital service use	3	0.70	0.555	**3**	**2.86**	**0.036**
Help required to prepare meals/meal delivery	1	0.16	0.686	1	0.26	0.608
Impairment in activities of daily living	3	2.37	0.069	**3**	**2.78**	**0.040**
Chair rise time	3	0.46	0.710	**3**	**3.98**	**0.008**
Life Space Index score	2	0.21	0.812	2	0.38	0.687

Bolded terms indicate statistical significance at *p* < 0.05

**Table 5:** Multivariable linear regression assessing covariates associated with change in SCREEN-8 score stratified by baseline nutrition risk status

		Not at risk (*n* = 3570)				At high risk (*n* = 1461)			
Parameter		Estimate	Standard error	95% confidence interval		Estimate	Standard error	95% confidence interval	
Intercept		− 0.06	0.47	− 0.97	0.86	**3.45**	**1.11**	**1.28**	**5.61**
Sex	Female	**− 0.57**	**0.24**	**− 1.04**	**− 0.10**	− 0.48	0.51	− 1.48	0.52
	Male	Reference				Reference			
Household income	Decrease from baseline	− 0.02	0.35	− 0.71	0.68	**1.94**	**0.63**	**0.71**	**3.18**
	Increase from baseline	0.30	0.28	− 0.24	0.84	0.69	0.68	− 0.64	2.02
	No change	Reference				Reference			
Marital status	Any change	− **1.52**	**0.62**	− **2.74**	− **0.30**	− 1.30	0.91	− 3.09	0.49
	No change	Reference				Reference			
Living alone	Yes/No	0.32	0.95	− 1.54	2.17	− 0.72	1.61	− 3.88	2.45
	No/Yes	− **1.98**	**0.72**	− **3.40**	− **0.56**	0.62	1.20	− 1.73	2.96
	Yes/Yes	− 0.44	0.32	− 1.08	0.20	0.23	0.49	− 0.74	1.20
	No/No	Reference				Reference			
Current smoker	Yes	− **1.83**	**0.81**	− **3.42**	− **0.24**	− 1.56	0.92	− 3.36	0.24
	No	Reference				Reference			
Self-rated hearing	Increase from baseline	0.36	0.28	− 0.19	0.91	0.43	0.53	− 0.62	1.47
	Decrease from baseline	0.06	0.22	− 0.38	0.50	− **1.02**	**0.47**	− **1.94**	− **0.09**
	No change	Reference				Reference			
Mental health condition	No/Yes	0.43	0.78	− 1.09	1.96	0.35	1.03	− 1.68	2.38
	Yes/No	− 1.12	0.65	− 2.39	0.15	− **3.07**	**1.24**	− **5.50**	− **0.64**
	Yes/Yes	− **0.83**	**0.31**	− **1.44**	− **0.22**	0.28	0.51	− 0.73	1.29
	No/No	Reference				Reference			
Gastrointestinal condition	Diagnosed at baseline	0.17	0.29	− 0.41	0.75	− **1.10**	**0.56**	− **2.19**	− **0.01**
	New diagnosis at follow-up	0.70	0.61	− 0.50	1.90	− **2.20**	**0.88**	− **3.93**	− **0.46**
	No/No	Reference				Reference			
Kidney condition	Yes at any point	− **1.51**	**0.68**	− **2.81**	− **0.21**	− 0.20	0.96	− 2.09	1.68
	No/No	Reference				Reference			
Pain-free rating^b^	No/No	0.12	0.30	− 0.47	0.70	− 0.55	0.56	− 1.65	0.55
	No/Yes	0.002	0.29	− 0.56	0.56	1.09	0.65	− 0.19	2.36
	Yes/No	0.21	0.34	− 0.45	0.88	− 1.48	0.80	− 3.05	0.09
	Yes/Yes	Reference				Reference			
Oral health problems	No/Yes	− 0.29	0.29	− 0.86	0.28	− 0.42	0.59	− 1.58	0.74
	**Yes/No**	0.05	0.31	− 0.56	0.67	**1.60**	**0.74**	**0.15**	**3.05**
	**Yes/Yes**	− **0.92**	**0.27**	− **1.44**	− **0.39**	− 0.02	0.58	− 1.17	1.12
	No/No	Reference				Reference			
Dentist visit	**No/No**	− **0.91**	**0.36**	− **1.62**	− **0.20**	− 0.92	0.66	− 2.21	0.37
	**No/Yes**	− 0.67	0.50	− 1.66	0.32	− **1.73**^**a**^	**0.74**	− **3.19**	− **0.27**
	**Yes/No**	− **1.32**	**0.58**	− **2.45**	− **0.19**	− 0.43	1.27	− 2.93	2.07
	Yes/Yes	Reference				Reference			
Psychologist/social service use	**Yes at any point**	− **1.25**	**0.45**	− **2.13**	− **0.37**	− 1.00	0.77	− 2.50	0.50
	No/No	Reference				Reference			
Hospital service use	No/Yes	− 0.44	0.32	− 1.06	0.18	− 0.65	0.55	− 1.72	0.43
	**Yes/No**	0.03	0.30	− 0.56	0.61	**1.35**	**0.63**	**0.12**	**2.58**
	Yes/Yes	− 0.04	0.38	− 0.79	0.71	− 0.48	0.73	− 1.92	0.95
	No/No	Reference				Reference			
Impairment with activities of daily living	**No/Yes**	− **1.02**^**a**^	**0.42**	− **1.85**	− **0.20**	− 0.65	0.69	− 2.01	0.71
	Yes/No	0.46	0.53	− 0.58	1.50	0.04	0.76	− 1.44	1.53
	Yes/Yes	− 0.28	0.61	− 1.47	0.91	− **2.56**	**0.92**	− **4.36**	− **0.77**
	No/No	Reference				Reference			
Chair rise time	**≤15 s/>15 s (declined)**	− 0.35	0.37	− 1.08	0.38	− **1.98**	**0.69**	− **3.33**	− **0.63**
	>15 s/≤15 s (improved)	− 0.03	0.29	− 0.60	0.55	− 0.48	0.70	− 1.85	0.90
	>15 s/>15 s (stable poor)	− 0.25	0.29	− 0.81	0.31	0.49	0.50	− 0.49	1.46
	≤ 15 s/≤15 s (stable adequate)	Reference				Reference			

When a “/” is used to separate baseline and follow-up measure. For example, “No/Yes” indicates that the variable of interest was not present at baseline, but present at follow-up. This regression model also adjusted for age, education level, polypharmacy, body mass index, Physical Activity Scale for the Elderly score, Social Support Survey score, problems with smell, problems with taste, lacking companionship, diagnosis of a dementia/neurological condition, cancer, cardiovascular condition, osteoporosis, endocrine condition, arthritis, respiratory condition, incontinence, recent surgery, self-rated vision, general practitioner/family physician visit, allied healthcare use, ophthalmologist/optometrist visit, alcohol intake frequency, help required to prepare meals/meal delivery, Life Space Index score, multimorbidity, and caregiver status

Bolded terms are statistically significant at *p* < 0.05

^a^Overall model effect is not significant at *p* < 0.05

^b^Overall effect significant at *p* < 0.05, but not with shown referent group

#### Participants not at high nutrition risk at baseline

Females experienced a decrease in SCREEN-8 score by 0.57 points as compared to males (CI [−1.04,  −0.10]). Participants who currently smoked experienced a decrease of 1.83 points compared to non-smokers (CI [−3.42,  −0.24]). Those who reported having a mental health condition at both baseline and follow-up experienced a decrease in SCREEN-8 by 0.83 points compared to participants who did not report having a mental health condition at any time (CI [−1.44,  −0.22]). Similarly, reporting a diagnosis of a kidney condition at baseline or follow-up was associated with a decrease in SCREEN-8 score by 1.51 points (CI [−2.81,  −0.21]). Participants who reported using psychologist or social services in the past 12 months at either baseline or follow-up experienced a decrease in SCREEN-8 score by 1.25 points compared to participants who did not report using these services (CI [−2.13,  −0.37]). Participants who did not report going to the dentist at baseline or follow-up or only went to the dentist at baseline experienced a decrease in SCREEN-8 score by 0.91 and 1.32 points, respectively, when compared to participants who visited the dentist at both timepoints (CI [−1.62,  −0.20], [−2.45,  −0.19]). Similarly, participants who consistently experienced oral health problems at baseline and follow-up experienced a decrease in SCREEN-8 by 0.92 points compared to participants who did not report any oral health problems (CI [−1.44,  −0.39]). Experiencing any change in marital status (e.g., single to widowed, or separated to married) was associated with a decrease in SCREEN-8 score by 1.52 points compared to participants who did not experience a change in marital status (CI [−2.74,  −0.30]). Newly living alone at follow-up and not at baseline was also significantly associated with a decrease in SCREEN-8 score by 1.98 points compared to participants who did not live alone at either timepoint (CI [−3.40,  −0.56]).

#### Participants at high nutrition risk at baseline

Gastrointestinal conditions were associated with SCREEN-8 score, with participants who reported a condition at baseline or a new condition at follow-up experiencing a decrease by 1.10 and 2.20 points, respectively, when compared to participants who did not report gastrointestinal conditions at baseline or follow-up (CI [−2.19. −0.01], [−3.93, −0.46]). Change in experiencing pain was associated with a change in SCREEN-8 score (*F* = 3.89, *p* = 0.009); however, significant differences between groups were not apparent when participants who were usually pain free at baseline and follow-up were used as the reference group. Participants who reported a decrease in hearing at follow-up experienced a 1.02 point decrease in SCREEN-8 score (CI [−1.94,  −0.09]). Hospital service use in the last 12 months was associated with a change in SCREEN-8 score, where participants who reported going to the hospital at baseline but not follow-up experienced a significant increase in SCREEN-8 score by 1.35 points compared to participants who did not report going to the hospital at either timepoint (CI [0.12, 2.58]). Participants who reported a decrease in their income experienced an increase in SCREEN-8 score by 1.94 points compared to participants who did not report a change in their income (CI [0.71, 3.18]). Participants who experienced impairment with their activities of daily living at both baseline and follow-up experienced a decrease in SCREEN-8 score by 2.56 points compared to participants who did not experience any impairment at baseline and follow-up (CI [−4.36,  −0.77]). Those who declined in their chair-rise performance between baseline and follow-up also experienced a decrease in SCREEN-8 score by 1.98 points compared to participants who performed adequately at baseline and follow-up (CI [−3.33,  −0.63]). Last, older adults who experienced oral health problems at baseline but not follow-up experienced an increase in SCREEN-8 score by 1.60 points compared to participants who did not experience oral health problems at either timepoint (CI [0.15, 3.05]).

## Discussion

High nutrition risk was common in this sample (29.0% at baseline and 35.6% at follow-up) and although most participants did not change their nutrition risk level between baseline and follow-up, more participants were likely to decline than improve in their nutrition. Decline was specifically seen in those not at high risk at baseline, whereas an increase in mean SCREEN-8 score was seen for those with high risk at baseline, suggesting potential impact of interventions or other changes that improved nutrition risk for some in this group.

This longitudinal analysis for the first time distinguished the determinants of a decline in nutrition for those not at risk at baseline (i.e., early in risk trajectory) and those who were initially at risk. Determinants were different among these groups but generally in anticipated directions. Poor oral health, lack of dental care, poor mental health, use of psychological or social services, and change in marital status or living arrangements were associated with a decline in SCREEN-8 scores (i.e., more nutrition risk) in those who were not at risk at baseline. These factors have previously been shown to be important determinants of nutrition risk or malnutrition (Bardon et al., 2021; Keller, 2007; Streicher et al., 2018; Thompson Martin et al., 2006). Smokers, females, and those with a kidney condition at either timepoint were also more likely to have declining SCREEN-8 scores. Alternatively, those participants at high nutrition risk at baseline had further declines in their SCREEN-8 scores and thus declining nutrition if they had a past or new gastrointestinal condition (of note the negative effect was greater for a new diagnosis at follow-up), decline in self-reported hearing from baseline, any impairment with activities of daily living at baseline or follow-up, and a decline in chair rise performance at follow-up. Decline in function has been associated with risk or malnutrition in previous work (Bardon et al., 2021; Keller, 2007; Thompson Martin et al., 2006; O’Keeffe et al. 2019), and points towards a frailer older adult continuing to decline in their overall health and nutrition. Three factors were associated with an improvement in SCREEN-8 scores in those at high risk at baseline: hospitalization in the 12 months prior to baseline but no hospitalization in the 12 months prior to follow-up, a decrease in income from baseline, and oral health problems at baseline that were resolved at follow-up. Hospitalization is known to be associated with high nutrition risk (Streicher et al., 2018) and could have offered an opportunity to identify malnutrition (Allard et al., 2016) and provide supports that could improve nutritional status. A declining income was associated with better nutrition at follow-up and could be attributed to increased social assistance programming or eligibility for specific benefits that supported nutrition, such as food-based social services. As we adjusted for living alone (26.0% at baseline) vs. with others (64.3% living with at least one other person with only 9.7% living with two or more people) in the analysis, any change in income due to a change in living with others would be accounted for in the regression. Further, as all participants were over the age of 65 years, new retirement was less likely to be a reason for this change in income. As sufficiency of income was not ascertained, we cannot further speculate on why a reported lower income was associated with higher SCREEN-8 scores at follow-up for those at nutrition risk at baseline. Meanwhile, improved oral health could have resolved challenges with eating that led to high nutrition risk at baseline.

This analysis demonstrates that declines in SCREEN-8 scores in those not at risk at baseline are associated with indirect factors like mental health and living situation, while those already at nutrition risk at baseline who had declines in SCREEN-8 scores had determinants consistent with frailty and worsening health. This analysis has confirmed the nutrition risk conceptualization (Keller, 2007) of indirect determinants that may not necessarily result in overt malnutrition and/or its effects and the view that several determinants are based on aging processes (Bardon et al., 2021). The DoMAP model also segments determinants by how direct their effect may be on three mechanisms for malnutrition. Several of the items on SCREEN-8 are included in the DoMAP as factors most directly associated with malnutrition through the mechanism of poor food intake (e.g., swallowing problems, poor appetite), but other direct factors on DoMAP include impaired activities of daily living including shopping and cooking (Volkert et al., 2019). Our findings on factors associated with a decline in SCREEN-8 in those already at nutrition risk at baseline are consistent with the DoMAP model. Indirect factors on the DoMAP model include living situation, mental health, health conditions, and poor oral health, which in our analysis were associated with declines in SCREEN-8 scores for those not at risk at baseline. Thus, this analysis of SCREEN-8 stratified by baseline nutrition risk also provides evidence for the consensus-developed DoMAP model of direct and indirect factors leading to malnutrition.

Use of preventive services like meal programs was very low in this sample (11.4% reported receiving help at some point) and we could not model change in professional or non-professional meal preparation or delivery help. However, other services, such as allied healthcare visits, were insignificant in both models. Based on the prevalence of nutrition risk and declines in SCREEN-8 scores seen over time, it can be surmised that there may be an underutilization as well as lack of availability of programs and services that could address low food intake and nutrition risk in community-living older Canadians (Keller et al., 2021). Hospital use and improved oral health resulted in higher SCREEN-8 scores for the high-risk group, suggesting that improvements are possible. However, lack of service use points to the need to operationalize primary care nutrition risk screening to find and treat the determinants of nutrition risk. If nutrition risk screening cannot be a global activity for all of those over the age of 65 in primary care to promote healthy aging, then targeted screening should be considered (Laur & Keller, 2017; Lee et al., 2018). Older adults experiencing life transitions such as a change in living situation or marital status, and those with mental health conditions and oral health problems should be screened for nutrition risk in primary care settings. Older adults with impaired activities of daily living and chair rise performance, as well as gastrointestinal diseases are likely already at high nutrition risk and prone to further declines in nutritional health, necessitating the monitoring and intervention of a dietitian (Keller et al., 2021; Laur & Keller 2017; Lee et al., 2018).

### Limitations of the study

This study is not without its limitations. CLSA is not representative of all Canadians, due to the exclusion of some groups (e.g., those who could not speak English or French), poor representation of certain demographics (e.g., race/ethnicity; 96% of the 5031 participants self-identified as white), and previous analyses have identified that participants in the CLSA comprehensive cohort are more educated and have higher household incomes compared to the Canadian population (Raina et al., 2019). Further, the comprehensive CLSA cohort was used for this analysis and was limited to those who could readily visit the urban academic test centres, further under-representing Canadians who live in small urban and rural communities. All data (except for body mass index) were collected by self-report questionnaires and due to missing data, our analyses were based on less than half of the eligible cohort sample and differences in demographic variables like sex, age, and education were detected when the cohort with missing data and the analytic cohort were compared. Finally, as with all longitudinal studies, loss to follow-up (*n* = 2332 of the original sample) occurred. Thus, the associations identified in this analysis should be considered as potentially biased by the data collection measures and sample constraints. Prior research has demonstrated lack of association in meta-analyses (Streicher et al., 2018) and systematic reviews (O’Keeffe et al., 2019), potentially due to similar measurement and sample issues.

## Conclusion

Nutrition risk is common in community-living older adults. Determinants of decline in nutrition over a 3-year period were dependent on whether the older adult was at nutrition risk at baseline. Indirect factors that may be effectively detected and managed through secondary prevention measures (e.g., oral health problems), some sociodemographic factors (e.g., living alone), and healthcare service use (e.g., not visiting a dentist) are associated with declines in nutrition for those not already at high nutrition risk, while those at high risk see further declines in their nutrition when functional changes and gastrointestinal disease are present. Implementation of nutrition screening in primary care, like the consensus-based nutrition care pathway for community-dwelling persons ages  ≥65 (Keller et al., 2021), could provide opportunities for individualized monitoring and timely intervention while accounting for older adults’ current nutritional status. Targeted nutrition risk screening, assessment, and treatment are needed to promote healthy aging for older Canadians.

## Contributions to knowledge

What does this study add to existing knowledge?

- This study demonstrates that determinants contributing to nutritional decline differ for those not at nutrition risk versus those at high risk.
- Indirect determinants of nutrition risk like living alone and smoking, in addition to determinants that may more directly indicate frailty, like impaired activities of daily living, signal high nutrition risk and continued trajectory towards malnutrition.
- Hospitalization and improved oral health can improve nutrition in high-risk older adults.

What are the key implications for public health interventions, practice, or policy?

- This analysis differentiates segments of the older adult population for nutrition risk screening and intervention in primary care.
- Older adults at high nutrition risk should have dietitian intervention and community supports to improve their food intake due to likely challenges with function.
- Older adults not yet at high nutrition risk can see declines in nutrition if they are also experiencing a life transition, mental health condition, or oral health problems.
- Nutrition risk screening and identification of supports to mitigate challenges are needed for older adults. SCREEN-8 is ideal for nutrition risk screening in primary care for early detection and timely intervention.

## Supplementary Information

Below is the link to the electronic supplementary material.

Supplementary file1 (PDF 284 KB)

Supplementary file2 (PDF 114 KB)

Supplementary file3 (PDF 211 KB)

## Data Availability

Data are available from the Canadian Longitudinal Study on Aging (www.clsa-elcv.ca) for researchers who meet the criteria for access to de-identified CLSA data.
