# Supplementary material for: Determinants of a decline in a nutrition risk measure differ by baseline high nutrition risk status: targeting nutrition risk screening for frailty prevention in the Canadian Longitudinal Study on Aging (CLSA)
Source: Can J Public Health. 2023 Mar 22;114(4):593–612. doi: 10.17269/s41997-023-00745-w (PMC10349003; doi:10.17269/s41997-023-00745-w)
Supplement: Supplementary file 1 — Supplementary file1 (PDF 284 KB) [file 41997_2023_745_MOESM1_ESM.pdf]

Supplementary Table 1: Descriptive Statistics for all Demographic, Health, Function and Service Use Measures for All Participants and Stratified by Baseline Nutrition Risk Status

Variable		All (n = 5031) n	All (n = 5031) %	Not at Risk (n = 3570) n	Not at Risk (n = 3570) %	At High Risk (n = 1461) n	At High Risk (n = 1461) %	Rao-Scott Chi-Square
Sex	Female	2252	44.8	1537	43.1	715	48.9	**χ^2 = 8.87**
	Male	2779	55.2	2033	56.9	746	51.1	**p = 0.003**
Age	65-69	2062	41.0	1467	41.1	595	40.7	
	70-74	1306	26.0	931	26.1	375	25.7	χ^2 = 2.84
	75-79	1154	22.9	809	22.7	345	23.6	p = 0.420
	≥80	509	10.1	363	10.2	146	10.0	
Body mass index	Low	284	94.4	207	5.8	77	5.3	χ^2 = 0.09
	Adequate	4747	5.7	3363	94.2	1384	94.7	p = 0.770
Current Smoker	No	4802	95.5	3466	97.1	1336	91.4	**χ^2 = 19.14**
	Yes	229	4.5	104	2.9	125	8.6	**p < 0.001**
Polypharmacy	<5 medications	2440	48.5	1841	51.6	599	41.0	**χ^2 = 23.53**
	≥5 medications	2591	51.5	1729	48.4	862	59.0	**p < 0.001**
Physical Activity Scale for the Elderly score	0 – 72.00	913	18.1	589	16.5	324	22.2	
	>72.00 – 110.72	1203	23.9	840	23.5	363	24.9	**χ^2 = 31.56**
	>110.72 – 148.43	1368	27.2	968	27.1	400	27.4	**p < 0.001**
	>148.43	1547	30.7	1173	32.9	374	25.6	
Problems with smell	No	4419	87.8	3176	89.0	1243	85.1	**χ^2 = 7.10**
	Yes	612	12.2	394	11.0	218	14.9	**p = 0.008**
Problems with taste	No	4723	93.9	3394	95.1	1329	91.0	**χ^2 = 12.37**
	Yes	308	6.1	176	4.9	132	9.0	**p < 0.001**
Chair rise time	≤15s / ≤15s (stable adequate)	2726	54.2	1989	55.7	737	50.4	
	≤15s / >15s (decline)	621	12.3	425	11.9	196	13.4	**χ^2 = 11.20**
	>15s / ≤15s (improved)	763	15.2	540	15.1	223	15.3	**p = 0.011**
	>15s / >15s (stable poor)	921	18.3	616	17.3	305	20.9	
Life Space Index Score	No change	1202	23.9	862	24.1	340	23.3	χ^2 = 0.98
	Increase from baseline	1587	31.5	1112	31.2	475	32.5	p = 0.612
	Decrease from baseline	2242	44.5	1596	44.7	646	44.2	
Oral health problems	No/No	1595	31.7	1222	34.2	373	25.5	
	No/Yes	953	18.9	692	19.4	261	17.9	**χ^2 = 25.46**
	Yes/No	879	17.5	620	17.4	259	17.7	**p < 0.001**
	Yes/Yes	1604	31.9	1036	29.0	568	38.9	

Multimorbidity	<2 conditions/<2 conditions	740	14.7	592	16.6	148	10.1	**χ^2 = 40.25** **p < 0.001**
	<2 conditions /≥2 conditions	139	2.8	106	3.0	33	2.3	
	≥2 conditions /<2 conditions	456	9.1	344	9.6	112	7.7	
	≥2 conditions /≥2 conditions	3696	73.5	2528	70.8	1168	80	
Dementia/neurological condition	No/No	4856	96.5	3460	96.9	1396	95.6	χ^2 = 1.39 p = 0.238
	Yes at any point	175	3.5	110	3.1	65	4.5	
Mental health condition	No/No	4037	80.3	2968	83.1	1069	73.2	**χ^2 = 39.71** **p < 0.001**
	No/Yes	130	2.6	80	2.2	50	3.4	
	Yes/No	123	2.4	80	2.2	43	2.9	
	Yes/Yes	741	14.7	442	12.4	299	20.5	
Cancer	No/No	3640	72.3	2593	72.6	1047	71.7	χ^2 = 2.68 p = 0.262
	Diagnosed at baseline	1076	21.4	744	20.8	332	22.7	
	New diagnosis at follow-up	315	6.3	233	6.5	82	5.6	
Gastrointestinal condition	No/No	4046	80.4	2933	82.2	1113	76.2	**χ^2 = 6.83** **p = 0.033**
	Diagnosed at baseline	779	15.5	505	14.1	274	18.8	
	New diagnosis at follow-up	206	4.1	132	3.7	74	5.1	
Cardiovascular condition	No/No	1854	36.9	1397	39.1	457	31.3	**χ^2 = 26.15** **p < 0.001**
	Diagnosed at baseline	2833	56.3	1936	54.2	897	61.4	
	New diagnosis at follow-up	344	6.8	237	6.6	107	7.3	
Osteoporosis	No/No	4161	82.7	2986	83.6	1175	80.4	χ^2 = 5.32 p = 0.070
	Diagnosed at baseline	658	13.1	451	12.6	207	14.2	
	New diagnosis at follow-up	212	4.2	133	3.7	79	5.4	
Endocrine condition	No/No	3164	62.9	2309	64.7	855	58.5	**χ^2 = 6.45** **p = 0.040**
	Diagnosed at baseline	1582	31.5	1076	30.1	506	34.6	
	New diagnosis at follow-up	285	5.7	185	5.2	100	6.8	
Arthritis	No/No	2460	48.9	1804	50.6	656	44.9	**χ^2 = 6.83** **p = 0.033**
	Diagnosed at baseline	2016	40.1	1381	38.7	635	43.5	
	New diagnosis at follow-up	555	11.0	385	10.8	170	11.6	
Respiratory condition	No/No	4172	82.9	3040	85.2	1132	77.5	**χ^2 = 39.88** **p < 0.001**
	Diagnosed at baseline	698	13.9	434	12.2	264	18.1	
	New diagnosis at follow-up	161	3.2	96	2.7	65	4.5	
Incontinence	No/No	3697	73.5	2718	76.1	979	67	**χ^2 = 25.20** **p < 0.001**
	Diagnosed at baseline	521	10.4	325	9.1	196	13.4	
	New diagnosis at follow-up	813	16.2	527	14.8	286	19.6	
Kidney condition	No/No	4777	94.9	3399	95.2	1378	94.3	χ^2 = 1.35 p = 0.245
	Yes at any point	254	5.1	171	4.8	83	5.7	
Surgery in the last 3 months	No/No	4512	89.7	3198	89.6	1314	89.9	χ^2 = 0.41

	Diagnosed at baseline	248	4.9	175	4.9	73	5.0	p = 0.816
	New diagnosis at follow-up	271	5.4	197	5.5	74	5.1	
Pain-free rating	No/No	919	18.3	559	15.7	360	24.6	**χ^2 = 34.24** **p < 0.001**
	No/Yes	739	14.7	513	14.4	226	15.5	
	Yes/No	497	9.9	333	9.3	164	11.2	
	Yes/Yes	2876	57.2	2165	60.6	711	48.7	
Self-rated hearing	No change	2213	44	1579	44.2	634	43.4	χ^2 = 0.54 p = 0.763
	Increase from baseline	883	17.6	606	17.0	277	19.0	
	Decrease from baseline	1935	38.5	1385	38.8	550	37.7	
Self-rated vision	No change	2232	44.4	1605	45	627	42.9	χ^2 = 3.53 p = 0.171
	Increase from baseline	1104	21.9	771	21.6	333	22.8	
	Decrease from baseline	1695	33.7	1194	33.4	501	34.3	
Self-rated general health	No change	2744	54.5	1993	55.8	751	51.4	χ^2 = 2.93 p = 0.231
	Increase from baseline	927	18.4	650	18.2	277	19.0	
	Decrease from baseline	1360	27.0	927	26.0	433	29.6	
Self rated mental health	No change	2659	52.9	1934	54.2	725	49.6	**χ^2 = 11.44** **p = 0.003**
	Increase from baseline	965	19.2	642	18.0	323	22.1	
	Decrease from baseline	1407	28.0	994	27.8	413	28.3	
Psychologist/social service use	No/No	4721	93.8	3384	94.8	1337	91.5	**χ^2 = 13.79** **p < 0.001**
	Yes at any point	310	6.2	186	5.2	124	8.5	
Allied health care use	No/No	2800	55.7	1987	55.7	813	55.7	χ^2 = 4.03 p = 0.260
	No/Yes	702	14.0	493	13.8	209	14.3	
	Yes/No	568	11.3	398	11.2	170	11.6	
	Yes/Yes	961	19.1	692	19.4	269	18.4	
General practitioner/family physician visit	No/no, no/yes, yes/no	495	9.8	373	10.5	122	8.4	**χ^2 = 4.52** **p = 0.036**
	Yes/yes	4536	90.1	3197	89.5	1339	91.7	
Ophthalmologist/optometrist visit	No/No	784	15.6	524	14.7	260	17.8	**χ^2 = 10.79** **p = 0.013**
	No/Yes	776	15.4	548	15.4	228	15.6	
	Yes/No	1031	20.5	739	20.7	292	20.0	
	Yes/Yes	2440	48.5	1759	49.3	681	46.6	
Dentist visit	No/No	532	10.6	339	9.5	193	13.2	**χ^2 = 31.87** **p < 0.001**
	No/Yes	371	7.4	232	6.5	139	9.5	
	Yes/No	156	3.1	98	2.7	58	4.0	
	Yes/Yes	3972	79.0	2901	81.3	1071	73.3	
Hospital service use	No/No	3156	62.7	2331	65.3	825	56.5	**χ^2 = 20.75** **p < 0.001**
	No/Yes	844	16.8	566	15.9	278	19.0	
	Yes/No	618	12.3	421	11.8	197	13.5	
	Yes/Yes	413	8.2	252	7.1	161	11.0	

Social Support Survey score	0 – 69.74	1046	20.8	582	16.3	464	31.8	**χ^2 = 109.92** **p < 0.001**
	>69.74 – 84.21	1379	27.4	956	26.8	423	29.0	
	>84.21 – 94.74	1253	24.9	946	26.5	307	21.0	
	>94.74	1353	26.9	1086	30.4	267	18.3	
Lacking companionship	Hardly ever	3695	73.4	2783	78	912	62.4	**χ^2 = 86.88** **p < 0.001**
	Some of the time	1113	22.1	685	19.2	428	29.3	
	Often	223	4.4	102	2.9	121	8.3	
Education level	Less than secondary school	339	6.7	212	5.9	127	8.7	**χ^2 = 16.17** **p = 0.001**
	Secondary school, no post-secondary education	485	9.6	339	9.5	146	10.0	
	Some post-secondary education	383	7.6	250	7.0	133	9.1	
	Post-secondary degree/diploma	3824	76	2769	77.5	1055	72.2	
Household income	No change	3937	78.2	2789	78.1	1147	78.5	χ^2 = 1.16 p = 0.561
	Decrease from baseline	515	10.2	369	10.3	146	10.0	
	Increase from baseline	580	11.5	412	11.5	168	11.5	
Marital status	No change	4801	95.4	3432	96.1	1369	93.7	χ^2 = 1.73 p = 0.188
	Any change	230	4.6	138	3.9	92	6.3	
Living alone	No/No	3506	69.7	2710	75.9	796	54.5	**χ^2 = 111.51** **p < 0.001**
	Yes/No	114	2.3	67	1.9	47	3.2	
	No/Yes	218	4.3	131	3.7	87	6.0	
	Yes/Yes	1193	23.7	662	18.5	531	36.3	
Alcohol intake frequency	No change	2666	53.0	1940	54.3	726	49.7	χ^2 = 4.03 p = 0.134
	Decrease from baseline	1189	23.6	806	22.6	383	26.2	
	Increase from baseline	1176	23.4	824	23.1	352	24.1	
Help required to prepare meals/meal delivery	No/No	4459	88.6	3206	89.8	1253	85.8	**χ^2 = 20.85** **p < 0.001**
	Yes at any point	572	11.4	364	10.2	208	14.2	
Activities of daily living	No/No	4177	83.0	3056	85.6	1121	76.7	**χ^2 = 32.81** **p < 0.001**
	No/Impairment	452	9.0	272	7.6	180	12.3	
	Impairment/No	197	3.9	124	3.5	73	5.0	
	Impairment/Impairment	205	4.1	118	3.3	87	6.0	
Caregiver status	No/No	1798	35.7	1296	36.3	502	34.4	χ^2 = 1.92 p = 0.589
	No/Yes	694	13.8	480	13.5	214	14.7	
	Yes/No	1114	22.1	809	22.7	305	20.9	
	Yes/Yes	1425	28.3	985	27.6	440	30.1	

When a “/” is used to separate baseline and follow-up measure. For example, “No/Yes” indicates that the variable of interest was not present at baseline, but present at follow-up. Bolded terms indicate statistical significance ($p < 0.050$) between the at risk and not at risk groups.
