# Supplementary material for: Determinants of a decline in a nutrition risk measure differ by baseline high nutrition risk status: targeting nutrition risk screening for frailty prevention in the Canadian Longitudinal Study on Aging (CLSA)
Source: Can J Public Health. 2023 Mar 22;114(4):593–612. doi: 10.17269/s41997-023-00745-w (PMC10349003; doi:10.17269/s41997-023-00745-w)
Supplement: Supplementary file 2 — Supplementary file2 (PDF 114 KB) [file 41997_2023_745_MOESM2_ESM.pdf]

Supplementary Table 2: Testing differences in baseline demographic variables between the eligible and analytic sample.

		Analytic Sample (n = 5031)		Sample with Missing Data		
		%	n	%	n	Rao-Scott Chi Square Test
Sex [a]	Female	44.76	2252	53.27	4048	**$\chi^2 = 56.49$ $p < 0.001$**
	Male	55.24	2779	46.73	3551	
Age [a]	65-69	40.99	2062	29.65	2253	**$\chi^2 = 165.94$ $p < 0.001$**
	70-74	25.96	1306	22.86	1737	
	75-79	22.94	1154	26.81	2037	
	≥80	10.12	509	20.69	1572	
Education level [b]	Less than secondary school	6.74	339	10.73	811	**$\chi^2 = 84.35$ $p < 0.001$**
	Secondary school, no post-secondary education	9.64	485	11.93	902	
	Some post-secondary education	7.61	383	8.65	654	
	Post-secondary degree/diploma	76.01	3824	68.69	5194	
Household income [c]	<$50,000	33.39	1680	46.95	3039	**$\chi^2 = 143.61$ $p < 0.001$**
	$50,000-$99,999	42.81	2154	36.70	2375	
	≥$100,000	23.79	1197	16.36	1059	
Marital status [d]	Single, never married or never lived with a partner	4.83	243	5.84	392	**$\chi^2 = 131.50$ $p < 0.001$**
	Married/living with a partner in a common-law relationship	68.57	3450	56.46	3793	
	Widowed	14.17	713	23.38	1571	
	Divorced/separate	12.42	625	14.32	962	
Living situation [e]	Alone	25.98	1307	29.89	2698	**$\chi^2 = 67.96$ $p < 0.001$**
	With one other person	64.34	3237	59.58	4168	
	With two or more people	9.68	487	10.53	716	
Nutrition Risk Status [f]	Not at Risk	70.96	3570	58.93	4017	**$\chi^2 = 99.29$ $p < 0.001$**
	At Risk	29.04	1461	41.07	2800	

[a] n = 7599 for sample with missing data

[b] n = 7561 for sample with missing data

[c] n = 6473 for sample with missing data

[d] n = 6718 for sample with missing data

[e] n = 7582 for nutrition risk for sample with missing data

[f] n = 6817 for sample with missing data
