# Supplementary material for: Determinants of a decline in a nutrition risk measure differ by baseline high nutrition risk status: targeting nutrition risk screening for frailty prevention in the Canadian Longitudinal Study on Aging (CLSA)
Source: Can J Public Health. 2023 Mar 22;114(4):593–612. doi: 10.17269/s41997-023-00745-w (PMC10349003; doi:10.17269/s41997-023-00745-w)
Supplement: Supplementary file 3 — Supplementary file3 (PDF 211 KB) [file 41997_2023_745_MOESM3_ESM.pdf]

Supplementary Table 3: Change in SCREEN-8 Score Estimates, Stratified by Baseline Nutrition Risk Status

		**Not at Risk (n = 3570)**				**At High Risk (n = 1461)**			
Parameter		Estimate	Standard Error	95% Confidence Interval		Estimate	Standard Error	95% Confidence Interval	
Intercept		-0.06	0.47	-0.97	0.86	**3.45**	**1.11**	**1.28**	**5.61**
Sex	Female	-0.57	0.24	-1.04	-0.10	-0.48	0.51	-1.48	0.52
	Male	Reference				Reference			
Age	70-74	**0.53**[a]	**0.25**	**0.05**	**1.02**	0.18	0.53	-0.86	1.21
	75-79	-0.03	0.30	-0.61	0.55	0.46	0.61	-0.73	1.65
	≥80	0.31	0.35	-0.37	0.98	-0.12	0.77	-1.63	1.40
	65-69	Reference				Reference			
Body mass index	Low	0.41	0.35	-0.28	1.10	-0.58	0.93	-2.40	1.23
	Adequate	Reference				Reference			
Education level	Less than secondary school	-0.11	0.35	-0.79	0.57	0.25	0.66	-1.04	1.54
	Secondary school, no post-secondary education	0.04	0.29	-0.53	0.62	0.36	0.58	-0.77	1.50
	Some post-secondary education	0.37	0.36	-0.33	1.07	0.40	0.58	-0.74	1.53
	Post-secondary degree/diploma	Reference				Reference			
Household income	Decrease from baseline	-0.02	0.35	-0.71	0.68	**1.94**	**0.63**	**0.71**	**3.18**
	Increase from baseline	0.30	0.28	-0.24	0.84	0.69	0.68	-0.64	2.02
	No change	Reference				Reference			
Marital status	Any change	**-1.52**	**0.62**	**-2.74**	**-0.30**	-1.30	0.91	-3.09	0.49
	No Change	Reference				Reference			
Living alone	Yes/No	0.32	0.95	-1.54	2.17	-0.72	1.61	-3.88	2.45
	No/Yes	**-1.98**	**0.72**	**-3.40**	**-0.56**	0.62	1.20	-1.73	2.96
	Yes/Yes	-0.44	0.32	-1.08	0.20	0.23	0.49	-0.74	1.20
	No/No	Reference				Reference			
Current smoker	Yes	**-1.83**	**0.81**	**-3.42**	**-0.24**	-1.56	0.92	-3.36	0.24
	No	Reference				Reference			
Alcohol intake frequency	Decrease from baseline	0.04	0.25	-0.44	0.53	-0.23	0.56	-1.33	0.87
	Increase from baseline	0.06	0.24	-0.41	0.54	-0.31	0.51	-1.30	0.69
	No change	Reference				Reference			
Polypharmacy	≥5 medications	0.14	0.22	-0.30	0.57	-0.38	0.49	-1.35	0.59

	<5 medications	Reference				Reference			
Multimorbidity	<2/≥2	0.21	0.90	-1.55	1.96	0.18	1.42	-2.61	2.97
	≥2/<2	-0.04	0.47	-0.95	0.87	0.93	0.88	-0.79	2.66
	≥2/≥2	-0.22	0.34	-0.89	0.46	-0.60	0.75	-2.07	0.88
	<2/<2	Reference				Reference			
Social Support Survey score	0 – 69.74	-0.10	0.32	-0.72	0.53	-0.28	0.62	-1.50	0.94
	>69.74 – 84.21	0.25	0.27	-0.28	0.78	-0.36	0.65	-1.63	0.92
	>84.21 – 94.74	0.35	0.28	-0.21	0.91	0.27	0.64	-0.98	1.53
	>94.74	Reference				Reference			
Lacking companionship	Some of the time	-0.18	0.28	-0.74	0.37	0.11	0.52	-0.91	1.14
	Often	0.19	0.62	-1.03	1.40	-0.74	0.70	-2.12	0.64
	Hardly ever	Reference				Reference			
Physical Activity Scale for the Elderly score	0 – 72.00	-0.44	0.0	-1.03	0.16	0.47	0.61	-0.72	1.67
	>72.00 – 110.72	-0.18	0.28	-0.73	0.37	0.43	0.61	-0.76	1.62
	>110.72 – 148.43	**-0.64a**	**0.28**	**-1.19**	**-0.09**	-0.41	0.60	-1.58	0.77
	>148.43	Reference				Reference			
Caregiver status	No/Yes	**0.68[a]**	**0.32**	**0.05**	**1.32**	0.80	0.63	-0.43	2.04
	Yes/No	0.19	0.25	-0.31	0.68	-0.20	0.62	-1.41	1.00
	Yes/Yes	0.10	0.28	-0.45	0.66	-0.52	0.53	-1.55	0.51
	No/No	Reference				Reference			
Problems with smell	Yes	-0.32	0.36	-1.02	0.39	-1.34	0.77	-2.84	0.16
	No	Reference				Reference			
Problems with taste	Yes	-0.17	0.49	-1.13	0.79	0.76	0.82	-0.85	2.37
	No	Reference				Reference			
Self-rated hearing	Increase from baseline	0.36	0.28	-0.19	0.91	0.43	0.53	-0.62	1.47
	Decrease from baseline	0.06	0.22	-0.38	0.50	**-1.02**	**0.47**	**-1.94**	**-0.09**
	No change	Reference				Reference			
Self-rated vision	Increase from baseline	-0.25	0.27	-0.78	0.28	-0.19	0.61	-1.37	1.00
	Decrease from baseline	**-0.49[a]**	**0.23**	**-0.94**	**-0.05**	-0.65	0.44	-1.52	0.22
	No change	Reference				Reference			
Self-rated general health	Decrease from baseline	0.15	0.30	-0.43	0.74	0.74	0.59	-0.41	1.89
	Increase from baseline	-0.15	0.25	-0.64	0.35	1.16	0.54	0.10	2.22
	No change	Reference				Reference			
Self-rated mental health	Decrease from baseline	-0.17	0.31	-0.78	0.43	-0.73	0.52	-1.75	0.28
	Increase from baseline	-0.32	0.23	-0.77	0.13	-0.67	0.50	-1.66	0.32
	No change	Reference				Reference			
Dementia/neurological condition	Yes at any point	-0.44	0.49	-1.40	0.53	-0.57	1.02	-2.56	1.43

	No/No		Reference				Reference		
Mental health condition	No/Yes	0.43	0.78	-1.09	1.96	0.35	1.03	-1.68	2.38
	Yes/No	-1.12	0.65	-2.39	0.15	**-3.07**	**1.24**	**-5.50**	**-0.64**
	Yes/Yes	**-0.83**	**0.31**	**-1.44**	**-0.22**	0.28	0.51	-0.73	1.29
	No/No		Reference				Reference		
Cancer	Diagnosed at baseline	0.06	0.25	-0.42	0.54	0.96	0.46	0.05	1.87
	New diagnosis at follow-up	-0.67	0.41	-1.48	0.13	0.86	0.83	-0.77	2.49
	No/No		Reference				Reference		
Gastrointestinal condition	Diagnosed at baseline	0.17	0.29	-0.41	0.75	**-1.10**	**0.56**	**-2.19**	**-0.01**
	New diagnosis at follow-up	0.70	0.61	-0.50	1.90	**-2.20**	**0.88**	**-3.93**	**-0.46**
	No/No		Reference				Reference		
Cardiovascular condition	Diagnosed at baseline	-0.22	0.25	-0.71	0.27	0.26	0.47	-0.67	1.19
	New diagnosis at follow-up	-0.40	0.38	-1.15	0.34	-0.13	0.75	-1.59	1.33
	No/No		Reference				Reference		
Osteoporosis	Diagnosed at baseline	0.01	0.35	-0.67	0.69	-0.61	0.64	-1.87	0.65
	New diagnosis at follow-up	0.18	0.54	-0.88	1.25	-0.13	0.90	-1.90	1.64
	No/No		Reference				Reference		
Endocrine condition	Diagnosed at baseline	-0.12	0.23	-0.57	0.33	1.05	0.49	0.08	2.02
	New diagnosis at follow-up	-0.45	0.53	-1.50	0.59	0.37	0.70	-1.00	1.73
	No/No		Reference				Reference		
Arthritis	Diagnosed at baseline	-0.20	0.24	-0.67	0.26	-0.24	0.56	-1.35	0.87
	New diagnosis at follow-up	-0.20	0.33	-0.84	0.45	-0.35	0.65	-1.63	0.93
	No/No		Reference				Reference		
Respiratory condition	Diagnosed at baseline	-0.72	0.38	-1.46	0.02	-0.50	0.55	-1.57	0.57
	New diagnosis at follow-up	-0.88	0.70	-2.24	0.49	0.33	0.98	-1.59	2.26
	No/No		Reference				Reference		
Incontinence	Diagnosed at baseline	-0.38	0.37	-1.11	0.34	0.76	0.72	-0.65	2.16
	New diagnosis at follow-up	0.25	0.30	-0.35	0.84	0.69	0.52	-0.33	1.71
	No/No		Reference				Reference		
Kidney condition	Yes at any point	**-1.51**	**0.68**	**-2.81**	**-0.21**	-0.20	0.96	-2.09	1.68

	No/No	Reference				Reference			
Surgery in the last 3 months	Surgery at baseline	-0.24	0.41	-1.05	0.57	-0.17	0.99	-2.12	1.78
	New surgery at follow-up	0.52	0.44	-0.34	1.38	0.54	0.78	-0.99	2.07
	No/No	Reference				Reference			
Pain-free rating [b]	No/No	0.12	0.30	-0.47	0.70	-0.55	0.56	-1.65	0.55
	No/Yes	0.002	0.29	-0.56	0.56	1.09	0.65	-0.19	2.36
	Yes/No	0.21	0.34	-0.45	0.88	-1.48	0.80	-3.05	0.09
	Yes/Yes	Reference				Reference			
Oral health problems	No/Yes	-0.29	0.29	-0.86	0.28	-0.42	0.59	-1.58	0.74
	Yes/No	0.05	0.31	-0.56	0.67	**1.60**	**0.74**	**0.15**	**3.05**
	Yes/Yes	**-0.92**	**0.27**	**-1.44**	**-0.39**	-0.02	0.58	-1.17	1.12
	No/No	Reference				Reference			
Dentist visit	No/No	**-0.91**	**0.36**	**-1.62**	**-0.20**	-0.92	0.66	-2.21	0.37
	No/Yes	-0.67	0.50	-1.66	0.32	**-1.73**[a]	**0.74**	**-3.19**	**-0.27**
	Yes/No	**-1.32**	**0.58**	**-2.45**	**-0.19**	-0.43	1.27	-2.93	2.07
	Yes/Yes	Reference				Reference			
Psychologist/social service use	Yes at any point	**-1.25**	**0.45**	**-2.13**	**-0.37**	-1.00	0.77	-2.50	0.50
	No/No	Reference				Reference			
Allied health care use	No/No	-0.19	0.30	-0.78	0.40	**1.41**[a]	**0.58**	**0.27**	**2.55**
	No/Yes	-0.10	0.33	-0.75	0.55	-0.11	0.63	-1.34	1.13
	Yes/No	0.07	0.28	-0.48	0.62	0.23	0.60	-0.96	1.42
	Yes/Yes	Reference				Reference			
General practitioner/family physician visit	No at any point	-0.47	0.37	-1.20	0.27	1.63	0.84	-0.01	3.28
	Yes/yes	Reference				Reference			
Ophthalmologist/optometrist visit	No/No	-0.45	0.30	-1.04	0.13	-0.68	0.65	-1.95	0.59
	No/Yes	-0.02	0.31	-0.62	0.58	0.32	0.57	-0.81	1.44
	Yes/No	0.36	0.26	-0.15	0.87	0.25	0.58	-0.89	1.39
	Yes/Yes	Reference				Reference			
Hospital service use	No/Yes	-0.44	0.32	-1.06	0.18	-0.65	0.55	-1.72	0.43
	Yes/No	0.03	0.30	-0.56	0.61	**1.35**	**0.63**	**0.12**	**2.58**
	Yes/Yes	-0.04	0.38	-0.79	0.71	-0.48	0.73	-1.92	0.95
	No/No	Reference				Reference			
Help required to prepare meals/meal delivery	Yes at any point	-0.15	0.38	-0.90	0.59	0.31	0.59	-0.86	1.47
	No/No	Reference				Reference			
Impairment with activities of daily living	No/Yes	**-1.02** [a]	**0.42**	**-1.85**	**-0.20**	-0.65	0.69	-2.01	0.71
	Yes/No	0.46	0.53	-0.58	1.50	0.04	0.76	-1.44	1.53

	Yes/Yes	-0.28	0.61	-1.47	0.91	**-2.56**	**0.92**	**-4.36**	**-0.77**
	No/No	Reference				Reference			
Chair rise time	≤15s / >15s (declined)	-0.35	0.37	-1.08	0.38	**-1.98**	**0.69**	**-3.33**	**-0.63**
	>15s / ≤15s (improved)	-0.03	0.29	-0.60	0.55	-0.48	0.70	-1.85	0.90
	>15s / >15s (stable poor)	-0.25	0.29	-0.81	0.31	0.49	0.50	-0.49	1.46
	≤15s / ≤15s (stable adequate)	Reference				Reference			
Life Space Index Score	Increase from baseline	-0.18	0.29	-0.74	0.39	-0.33	0.53	-1.36	0.71
	Decrease from baseline	-0.15	0.26	-0.66	0.36	-0.45	0.53	-1.48	0.59
	No change	Reference				Reference			

Note: When a "/" is used to separate baseline and follow-up measure. For example, "No/Yes" indicates that the variable of interest was not present at baseline, but present at follow-up. **Bolded terms are statistically significant at p < 0.050**

[a] Overall model effect is not significant at p < 0.050.

[b] Overall effect significant at p < 0.050, but not with shown referent group
